# Noninvasive Assessment of Early Dental Lesion Using a Dual-Contrast Photoacoustic Tomography

**DOI:** 10.1038/srep21798

**Published:** 2016-02-23

**Authors:** Renxiang Cheng, Jiaojiao Shao, Xiaoxiang Gao, Chao Tao, Jiuyu Ge, Xiaojun Liu

**Affiliations:** 1 Key Laboratory of Modern Acoustics, Department of Physics, Collaborative Innovation Center of Advanced Microstructures, Nanjing University, Nanjing, 210093, China; 2 Nanjing Stomatological Hospital, Medical School of Nanjing University, Nanjing, 210008, China

## Abstract

Dental hard tissue lesions, including caries, cracked-tooth, etc., are the most prevalent diseases of people worldwide. Dental lesions and correlative diseases greatly decrease the life quality of patients throughout their lifetime. It is still hard to noninvasively detect these dental lesions in their early stages. Photoacoustic imaging is an emerging hybrid technology combining the high spatial resolution of ultrasound in deep tissue with the rich optical contrasts. In this study, a dual-contrast photoacoustic tomography is applied to detect the early dental lesions. One contrast, named B-mode, is related to the optical absorption. It is good at providing the sharp image about the morphological and macro-structural features of the teeth. Another contrast, named S-mode, is associated with the micro-structural and mechanical properties of the hard tissue. It is sensitive to the change of tissue properties induced by the early dental lesions. Experiments show that the comprehensive analysis of dual-contrast information can provide reliable information of the early dental lesions. Moreover, the imaging parameter of S-mode is device-independent and it could measure tissue properties quantitatively. We expect that the proposed scheme could be beneficial for improving safety, accuracy and sensitivity of the clinical diagnosis of the dental lesion.

Dental hard tissue lesions, such as caries, cracked-tooth, and so on, are known as the most prevalent diseases of people worldwide^1^. According to up-to-date epidemiological investigation, there has been a remarkable increase in the prevalence of dental caries recently in all age range, including children, adults and elderly people^1^. Dental lesion will incur localized dissolution and destruction of calcified hard tissues, and further cause oral pain, tooth loss through pulp and periapical tissue inflammation^2^. Therefore, the dental lesion and correlative diseases greatly decrease the life quality of patients throughout their lifetime^2^,^3^.

Diagnosis of the early dental lesion has important clinical significance for the prevention and treatment of such diseases. If the lesions could be diagnosed at an initial stage, the progress of dental diseases can be stopped through preventive treatment, such as diet modification, plaque control, appropriate usage of fluoride for early caries and occlusal adjustment, adhesive crown restoration for cracked tooth. Otherwise, as the lesion progressing, destroy of the hard tissue could not be repaired unless employing aggressive treatment, such as filling treatment, root canal treatment and post-crown restoration. Even more, the involved tooth could not be preserved as root fracture or mass destruction.

Visual and radiographic examinations are the most widely used methods for dental disease diagnosis currently. However, they are inefficient in assessing the hard tissue lesion at an initial stage. Visual examination, as a subjective method, has a low reproducibility in detecting early enamel lesions, due to the dependence of the knowledge and clinical experience of the examiner. Radiographic examination is highly accurate for cavitated proximal lesions, but is poorly sensitive for non-cavitated lesions, such as white spot caries and cracks, which commonly appear at the early stage of dental lesion^3^,^4^. Researchers are still looking for advanced utility methods that can significantly improve the sensitivity and specificity for objective assessment of early lesions in the teeth^5^,^6^,^7^,^8^.

Photoacoustic tomography (PAT)^9^ is a hybrid non-invasive imaging modality combining the rich optical contrast with high ultrasonic resolution in turbid tissue. By extracting different imaging parameters from the photoacoustic signals, the PAT can effectively reflect the biochemical information^10^,^11^,^12^,^13^,^14^, biomechanical properties^15^,^16^,^17^, microstructural characteristics^18^,^19^,^20^,^21^,^22^,^23^,^24^,^25^, blood velocity^26^,^27^, temperature distribution^28^,^29^, and so on. Besides, non-ionizing laser used in PAT is much safer than the ionizing radiation, e.g., X-ray which as the radiographic method is used for the dental examination in clinics. Generally, rich contrasts and biosecurity make PAT have the natural advantages in mapping the physiological structure and function of biological tissue, such as breast cancer detecting^30^,^31^,^32^,^33^,^34^,brain imaging^35^,^36^, vessel diseases monitoring^28^,^37^,^38^, joint imaging^39^,^40^, etc^41^,^42^,^43^,^44^,^45^,^46^,^47^.

We hypothesized that PAT could non-invasively image the teeth structure and detect the early dental lesion. However, the current studies mostly focused on the soft tissue. Seldom applications have been done to the hard tissue, such as tooth. In this study, we devoted to imaging the human tooth cross-section and detecting the early lesion in the dental hard tissue by a dual-contrast PAT system. Two different parameters, intensity and spectral slope, have been extracted from the photoacoustic signals to form images, respectively. Intensity of photoacoustic signals is used as the imaging parameter in conventional PAT. Its brightness indicates the relative optical absorption in tissue, which is similar to the classic B-mode ultrasound imaging. We call this mode as B-mode PAT in our study. The spectral slope of photoacoustic signal is used as another imaging parameter. This contrast has been proved to qualify the microstructure in deep tissue effectively^18^,^19^ and be highly related to the stiffness properties^15^ of the tissue. We name this mode as S-mode PAT from the term “slope”.

In this study, we attempt to apply the dual-contrast PAT system to early dental hard tissue lesion detection and explore the reliability and sensitivity of the detection system.

## Results

### Materials and sample preparation

Both health teeth and lesion teeth were imaged by the B-mode and S-mode PAT. Fresh human teeth were extracted in an alveolar surgery clinic from the patients due to their oral health problems, including impacted third molar, supernumerary tooth, and the requirement of orthodontic treatment. Uses of these teeth are approved by patients and the Institutional Review Board of Institute of Stomatological hospital, Medical School of Nanjing University. The teeth were stored at 4 °C in formalin solution. During all subsequent procedures, the samples are either stored in water or kept moist to prevent shrinkage cracks from dehydration. Five samples are examined by an experienced dentist and photographed by the digital camera. These five samples are numbered as T1, T2, T3, T4, and T5.

We chose two samples (T4 and T5) with lesions from all the extracted teeth. One sample (T5) has a natural white spot enamel caries on the proximal surface. This early caries was not discovered by radiological examination before extraction. The artificial cracks were created on the other sample (T4) by dental forceps clamping to imitate cracked tooth. First, the roots of the teeth were embedded into self-curing acrylic resin and fixed on the holding device. Then a dental forcep was used, and the sharp beaks were placed on buccal and lingual surfaces, exerting pressure and squeezing the sample until cracks came out.

### PAT imaging system

**Figure 1:**
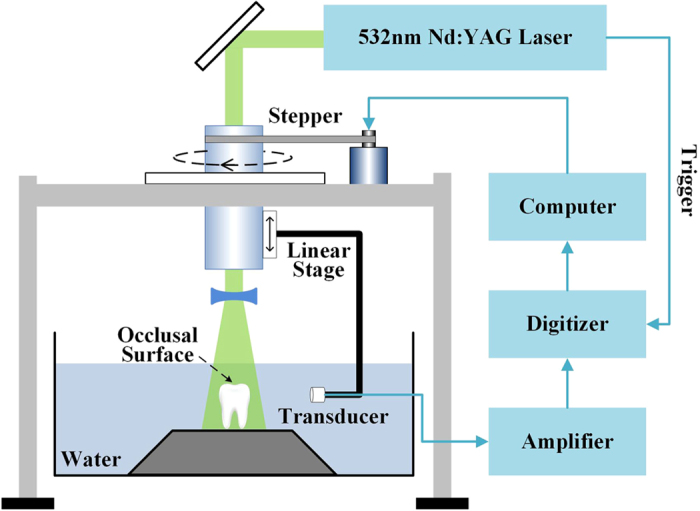
Schematic diagram of the experimental system. The laser irradiates the tooth occlusal surface vertically. Photoacoustic signals detected by a transducer are amplified by amplifier and recorded by a data-acquisition card. Driven by a stepper motor, the transducer scans circularly around the tooth with 120 steps. A linear stage is employed to control the position of the scanning surface in the *z*-axis. The system is controlled by a computer.

Figure 1 shows the imaging system. A Q-switched Nd:YAG laser with wavelength of 532 nm, pulse width of 8 ns, pulse reputation rate of 10 Hz and energy of 80 mJ was employed as the irradiation source. The laser spot with a radius of about 1.5 cm corresponds to the incident fluence on the sample surface of about 11 mJ/cm^2^ < 20 mJ/cm^2^ (the ANSI safety limit^48^), which is safe for the tooth sample^49^. Meanwhile, such laser pulse energy can provide an effective penetration depth of no less than 9 mm. This depth is deep enough to cover the crown of a tooth, where the early lesions often happen. The tooth root was vertically fixed in the water tank, and the laser beam irradiated the tooth on the occlusal surface along the long axis. Driven by a stepper motor, the transducer moved around the tooth with 120 steps in a circle for one complete scan. The stepper motor was controlled by computer. Unless otherwise specified, the ultrasound transducer (V310, Panametrics) with center frequency of 4.39 MHz and relative bandwidth of 100.2% at −6 dB was used for the PA signal detection. The detected photoacoustic signals were amplified (SA-230F5, NF) and recorded by a data-acquisition card (PCI-5105, NI) with sampling frequency of 60 MHz. The signals were averaged 40 times to reduce noise at each position. Both the tooth and ultrasound transducer were immersed in water to obtain good acoustic coupling. The scanning plane, i.e., imaging plane, was perpendicular to the long axis and parallel to the occlusal surface of tooth. A linear stage was employed to control the position of the scanning plane in the vertical direction.

### Imaging of Health tooth

**Figure 2:**
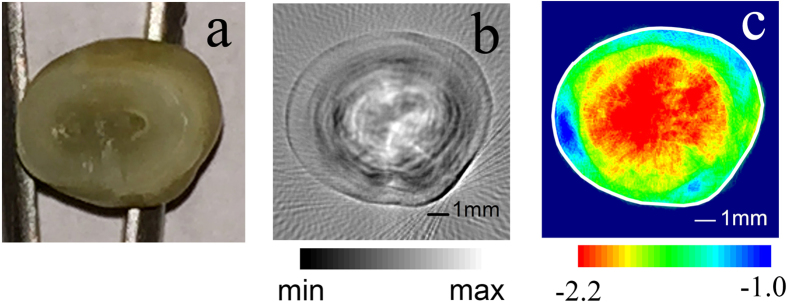
The images of a health tooth (T1) obtained in two modes. (**a**) The optical image of a tooth slice in the imaging plane. The part with a high optical transparency is enamel and low optical transparency is dentin with dental pulp, vascular and liquid. (**b**) The B-mode PAT image of tooth section. A layered concentric structure can be clearly observed. The inner layer has the relative large image value, which indicates strong optical absorption. The outer layer has the relative small image value, which means the weak optical absorption. (**c**) The S-mode image of the same section. A two-layer concentric structure can be identified in the S-mode PAT. The inner layer shows a small spectral slope and the outer layer has a large spectral slope. The solid white line is manually plotted according to the tooth outline in the B-mode image.

Figure 2 illustrates the images of one health tooth (T1). Figure 2(b) is the B-mode image of one cross-section of the tooth crown. The picture of the same tooth cross-section is shown in Fig. 2(a) for comparison. In Fig. 2(b), the B-mode PAT shows an accurate contour of the tooth. Moreover, layered concentric structure as the precise anatomy can be clearly observed in this B-mode PAT. The image value of the inner layer is higher, which indicates strong optical absorption. The image value of the outer layer is lower, which indicates weaker optical absorption. Meanwhile, the inner layer has the exact same size as the dentin structure and the outer layer has the exact same size as the enamel structure in this cross-section. The tooth crown consists of enamel and dentin. Enamel is the white, protective external surface of the anatomic crown. It has higher transparency and weaker optical absorption. Dentin is the yellowish tissue underlying the enamel making up the major bulk of the inner portion. The dentin involved pulp chamber filled with dental pulp, vascular, and liquid, has a low optical transparency and stronger optical absorption than the enamel^50^.

The S-mode image of the same cross-section in the health tooth is given in Fig. 2(c). A clear two-layer concentric structure also can be identified in the S-mode PAT. Spectral slope values of the two layers are different. The boundary between the two layers is obvious. The average spectral slope of the dentin layer is smaller than that of the enamel layer.

Both the B-mode and the S-mode images accurately reflect the anatomy of the health tooth crown. However, the B-mode contrast comes from the diversity of optical absorption^51^,^52^, whereas, the S-mode contrast is related to the microstructure and mechanical properties of the tooth^15^,^16^,^18^,^19^,^25^.

### Imaging of lesion tooth

**Figure 3:**
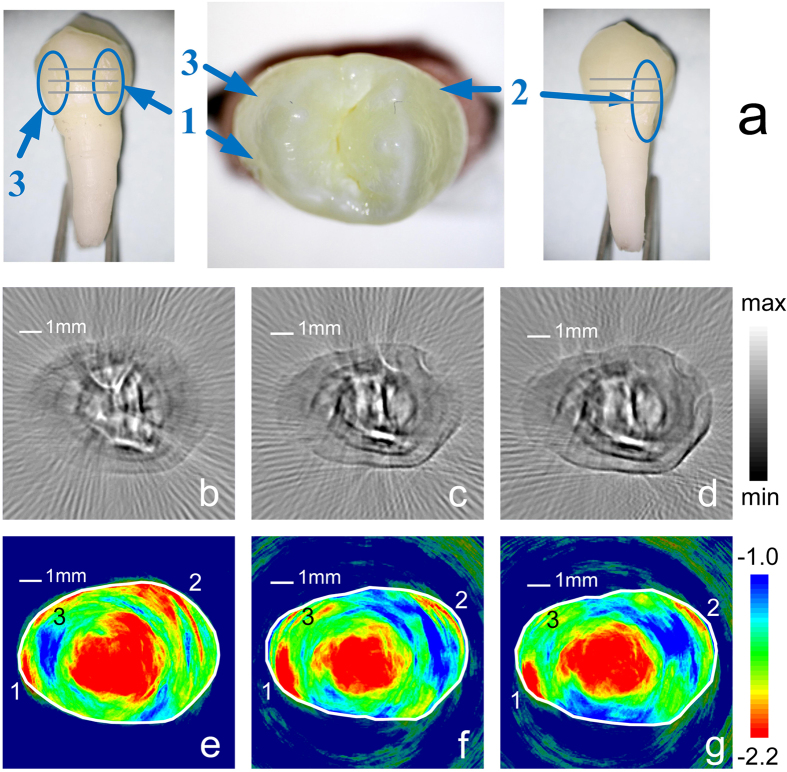
The PAT images of a lesion tooth (T4). The upper row (**a**) is the specimen with lesions, which are labeled from one to three. The position is shown. The middle row [(**b–d**)] and lower row [(**e–g**)] are B-mode images and S-mode images, respectively. For the middle and lower rows, the three columns from the left to the right are PAT images in three sections from top to bottom of the lesion tooth and the same column are the same section. The approximate positions of imaging section are marked in Fig.3(a) with the gray solid lines. For S-mode PAT images, several significant low slope regions break the annular region with the high slope. Comparing with the corresponding S-mode PAT and the optical image, the low slope regions correspond to the lesions in the tooth and are labeled as the same number. The solid white lines in the S-mode images are manually plotted according to the tooth outline in the corresponding B-mode images.

Lesion teeth are also imaged by the dual-contrast PAT. This sample (T4) shown in Fig. 3(a) is a maxillary first premolar donated by a 16 years old patient who needs to extract four first premolars for orthodontic treatment.

Figure 3(b–d) illustrates the B-mode images of three section from top to bottom. Their approximate positions are marked in Fig. 3(a) with the gray solid lines. The distance between two adjacent sections is 1.0 mm. In the B-mode images, the overall structure of this sample is similar to that of the normal tooth shown in Fig. 2(b). The pixels corresponding to the enamel have low image values, whereas, those corresponding to the dentin have relatively high values. The optical absorption of the dentin is stronger than the enamel. However, because of the individual differences of the teeth, some thick line patterns appear in the dentin of this sample (T4), but were not observed in the tooth (T1) shown in Fig. 2(b). These patterns shown in Fig. 3(b–d) could be related to the macro-structure in the dentin of this sample, as shown in the supplementary Fig. S1. In short, the B-mode contrast clearly shows the accurate shape, size, and macro-structure of the examined tooth cross-section, however, it fails to show significant contrast on the dental lesions.

Figure 3(e–g) shows the S-mode image of the lesion tooth. The discrepancy in the background noise could be related to the different imaging depth of Fig. 3(e–g). A tooth contour is shown in the S-mode images. Generally, the central region, where corresponds to the dentin, has small slope value. The margin, corresponding to the enamel, shows large slopes. These results agree with those of the normal teeth. However, the difference is that several significant low slope regions break the annular region with the high slope, which corresponds to the enamel. Comparing the S-mode PAT with the optical image, as shown in Fig. 3(a,e–g), it can be noticed that these low slope regions correspond to the lesions in the tooth. It is said that the lesions can be clearly identified in the S-mode PAT.

### Statistical analysis

The average spectral slope values of enamel and dentin were quantified. The statistical analysis was performed to examine the reliability of early dental lesion detection by the dual-contrast PAT system.

**Figure 4:**
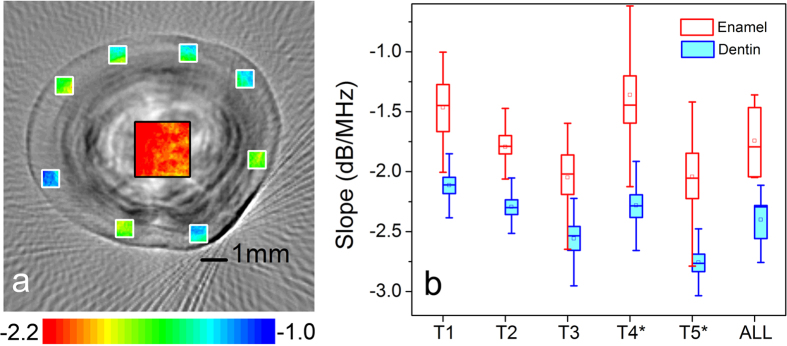
The spectral slope value of enamel and dentin of the teeth. (**a**) The spectral slope value of the enamel is estimated from randomly selected squares with a size of 0.6 mm × 0.6 mm shown as the white box. The slope value of the dentin is estimated from a square with a size of 2.3 mm × 2.3 mm shown as the black box. (**b**) The slope value of enamel and dentin of several specimens. For the lesion tooth [T4 and T5], the box selection excludes the lesion regions. For all specimens, the enamel has a spectral slope (mean ± SD) of −1.70 ± 0.34 dB/MHz and the dentin has a spectral slope (mean ± SD) of −2.45 ± 0.31 dB/MHz.

In the enamel layer, eight 0.6 mm × 0.6 mm data squares [the white windows in Fig. 4(a)] close to the out contour were evenly selected on each cross-section. For cracked teeth, the data squares needed to avoid lesion regions. The length and width of the data square are smaller than the enamel thickness, which confirms that all data comes from the enamel.

In the dentin layer, a 2.3 mm × 2.3 mm data square [black window in Fig. 4(a)] was applied in the central region of each cross-section. The entire data square should be in the dentin layer, without contacting the boundary and enamel layer.

For each sample, the spectral slopes [mean ± standard deviation (SD)] of the enamel are −1.52 ± 0.49 dB/MHz, −1.77 ± 0.30 dB/MHz, −2.12 ± 0.53 dB/MHz, −1.37 ± 0.75 dB/MHz, and −2.10 ± 0.69 dB/MHz, respectively. The spectral slopes (mean ± SD) of the dentin are −2.12 ± 0.27 dB/MHz, 2.28 ± 0.23 dB/MHz, −2.59 ± 0.36 dB/MHz, −2.29 ± 0.37 dB/MHz, −2.76 ± 0.28 dB/MHz, respectively. For all samples, the average spectral slopes (mean ± SD) of the enamel and dentin are −1.70 ± 0.34 dB/MHz, −2.45 ± 0.31 dB/MHz, respectively. The *t*-test confirms that the spectral slope of the enamel is significantly higher than it of the dentin (*p* < 0.001).

**Figure 5:**
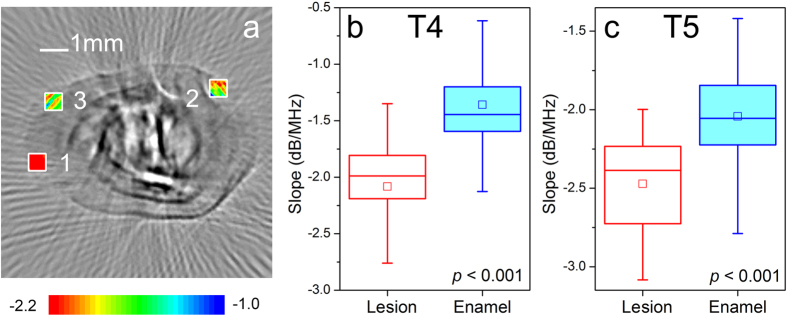
The spectral slope of the lesion region and the surround enamel. (**a**) The slope value of the lesion region is estimated from squares with a size of 0.6 mm × 0.6 mm shown as the black box. (**b**) The spectral slope value of the lesion specimen T4. (**c**) The spectral slope value of the lesion specimen T5. The *t*-test confirms the significantly statistic difference (p < 0.001).

Figure 5 compares the spectral slope of the lesion region with the surrounding normal enamel. It is seen that the spectral slope (mean ± SD) of the lesion region is about −2.19 ± 0.84 dB/MHz on average, which is significantly lower than the spectral slope (mean ± SD) 1.55 ± 0.96 dB/MHz of their surrounding enamel (*p* < 0.001). Therefore, the S-mode PAT can provide good contrast to identify the lesion in the enamel.

## Discussion

Both the B-mode PAT and S-mode PAT have been employed to image the health tooth and lesion tooth in this study. The images obtained by the two modes have different imaging parameters and their contrasts reflect the different physiological aspect of the tooth.

The B-mode PAT provides sharp images about the shape and structure of the teeth. The contrast of the B-mode PAT comes from the diversity of optical absorption in tissue. The image parameter of B-mode PAT image is proportional to the relative optical absorption coefficients. The B-mode images show that the outer layer of the tooth has a small image value than the inner layer. The outer layer of the tooth is enamel, which has a high optical transparency and weak light absorption. The inner layer is dentin, which has a low optical transparency and shows a color of light yellow. Moreover, dentin and pulp chamber are rich in dental pulp, blood vessel, and so on. Dental have relatively stronger optical absorption than enamel. Therefore, the B-mode images in Figs 2(b) and 3(b–d) correctly reveal the anatomical structure of the tooth according to their optical absorption discrepancy.

However, regretfully, the B-mode images are not sensitive to reflect the changes induced by the early enamel lesion, such as the mechanical damage and caries lesion discussed in this study. The lesion area does not show significantly contrast to the surrounding normal tissue in the reconstructed images. It could be the results of the following two reasons:

One reason could be that these lesions do not significantly change the characteristics of optical absorption. The early lesion could change the microstructural and mechanical characteristics of the enamel^53^,^54^,^55^, whereas, they could have little influence on the optical absorption. The contrast of the B-mode PAT depends on the optical absorption. Therefore, it could be insensitive in monitoring these lesions.

**Figure 6:**
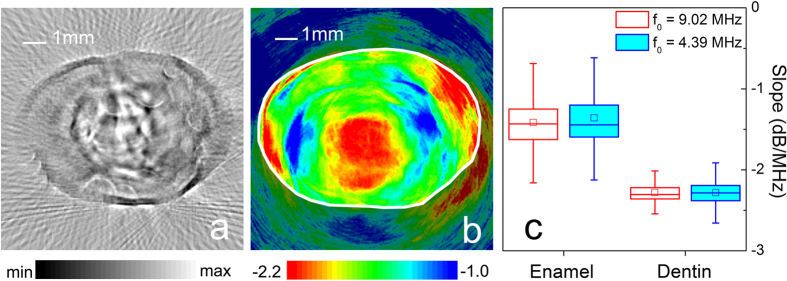
The PAT images obtained by the ultrasound transducer with a center frequency of 9.02 MHz and bandwidth of 10.65 MHz. (**a**) B-mode PAT image of the lesion tooth. (**b**) S-mode PAT image of the lesion tooth. The solid white line is manually plotted according to the tooth outline in the B-mode image. (**c**) The spectral slope value of the same specimen obtained by two ultrasound transducers, where the statistic results of *f*_0_ = 4.39 MHz and 9.02 MHz correspond to Figs 3(f) and 6(b), respectively.

Another reason could be related to the resolution of the B-mode PAT. The resolution of B-mode PAT in the imaging plane is *R*_*A*_ = 0.88 *c*/Δ*f*  9, where *c* is the speed of sound and Δ*f* is the bandwidth of ultrasound transducer. The transducer (V310, Panametrics) used in the above study has a bandwidth of 4.38 MHz, which corresponds to the resolutions of about 0.3 mm. The typical crack in enamel has a width which is less than 25 μm^6^,^56^, which is beyond the resolution limitation of the system. We have improved the resolution of B-mode PAT by replacing the ultrasound transducer with a high-frequency model. Figure 6 shows the image getting by the ultrasound transducer (V312, Panametrics) with a center frequency of 9.02 MHz and a bandwidth of 10.65 MHz, which corresponds to a resolution of about 0.12 mm. The B-mode PAT with a high resolution can reveal more details than the system with a low resolution. The tooth lesion could induce some random micro-cracks or structure changes in the surrounding enamel. These changes could be reflected by a high resolution system. Therefore, with the improvement of resolution, some random patterns appear in the lesion region, as shown in Fig. 6(a). However, these patterns are blurry and low contrast. They still cannot provide reliable imaging evidence of the existence of the lesion.

The S-mode PAT provides a good contrast for imaging the enamel lesion. The imaging parameter of the S-mode PAT is the spectral slope of PA signals. The slope value could be related to two properties of tissue at least. One is microscopic features in biological tissue. Similar to US spectrum analysis^57^, which has been used to discrimination of microscopic features in biologic tissue, photoacoustic spectral slope is also closely related the microstructural properties of the tissue^18^,^19^,^20^,^21^. The lower value of PA spectral slope usually indicates a relative larger characteristic size of microstructure in the tissue. Another property affecting spectral slope is the stiffness of target. An *et al.*^15^ revealed a close connection between power spectrum parameter and Young’s modulus of the target. A higher Young’s modulus corresponds to a wider bandwidth, which means a larger spectral slope of the signals.

Enamel is composed of tightly-packed rod unit. Dentin is porous, which consists of dentinal tubules and branching canalicular systems. Dentin has a microscopic feature with a big characteristic size than the enamel has. Moreover, the crystalline enamel is usually stiffer than the bone-like dentin^58^,^59^ and Young’s modulus of enamel is larger than dentin. Therefore, according to the relationship between the spectral slope and tissue properties, enamel should correspond to a larger spectral slope than dentin. The S-mode images of the teeth correctly reflect the difference of the physiological properties between enamel and dentin.

Lesion will change the properties of enamel. Lesion could result in some additional microstructure, such as cracks. Lesion tissue could be sparser than normal tissue^1^. Moreover, the enamel broken induced by the lesion decreases the effective stiffness of the enamel. These changes of physiological features will decrease the spectral slope corresponding to the lesions. Therefore, the S-mode PAT can clearly image the lesion in the enamel, as shown in Figs 3 and 6.

Additionally, the image value of S-mode PAT does not depend on the instrument and user operation parameters (e.g. laser power, amplifier gain, and so on) for imaging, after the easy calibration procedure. Figure 6(c) compares the spectral slope of the same specimen obtained by two imaging processes, where the statistic results of *f*_0_ = 4.39 MHz and 9.02 MHz correspond to Figs 3(f) and 6(b), respectively. It is seen that the obtained image parameters, i.e., spectral slope, are in a good agreement with each other, although they are obtained by two independent imaging processes with the different ultrasound transducers. Because of this merit of the spectral slope, the S-mode PAT could provide a device-independent and quantitative measurement of tissue properties. Therefore, the S-mode PAT could be helpful for an apples-to-apples comparison of images acquired by different researchers employing different instruments.

In summary, a dual-contrast PAT is proposed to detect the early lesion in the human tooth. The image contrast of B-mode PAT is related to the optical absorption, while the contrast of S-mode PAT is associated with the microstructural and mechanical properties of the specimen. Experiments show that the B-mode PAT is good at providing the sharp image about the morphological and macrostructural features of the teeth. The S-mode image is sensitive to the early lesion in the teeth.

Finally, it is noticed that B-mode PAT and S-mode PAT are not two separate imaging systems, but the two imaging modes of one PAT system. The two modes extract different image parameters from the same photoacoustic signals and provide dual-contrast for the same specimen. The two imaging modes can be easily combined to detect the tooth lesion without any extra tests or additional cost of hardware. Moreover, the images obtained by the two modes reflect the different physiological feature of the tooth. The comprehensive analysis of dual-contrast information from two PAT modes can provide reliable information of the early lesion of the teeth. Therefore, we can expect that PAT with the dual contrasts could be beneficial for improving safety, accuracy and sensitivity of the clinical diagnosis of the dental lesion.

## Methods

### Reconstruction of the B-mode image

The detected photoacoustic signals from each scanning cross-section of each tooth are processed in dual-contrast method.

The B-mode PAT images were reconstructed by modified delay-and-sum algorithm^60^,


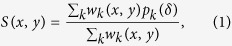


*S*(*x*, *y*) represents the image value at position (*x*, *y*), *p*_*k*_ is the PA signal recorded by the transducer element at the *k-*th scanning position, δ is the time delay of PA signal between the position (*x*, *y*) and the detector. *w*_*k*_(*x*, *y*) is the weighting factor, which takes into account the angular sensitivity of the transducer at the *k*-th scanning position and the image pixel at (*x*, *y*). In the B-mode PAT, the image values represent the relatively optical absorption.

### Reconstruction of S-mode images

The reconstruction of S-mode image can be briefly described as follows^18^. *p*_*k*_(*n*) indicates the PA signal at the *k*-th position. *n* represents the discrete time. i.e., *t* = *n*Δ*t* and Δ*t* is sampling interval. The power spectrum of the data segment {*p*_*k*_(*n–L*/2), …, *p*_*k*_(*n*),…, *p*_*k*_(*n* + *L*/2)} within a (*L* + 1)-point window is obtained by spectrum conversion. After spectrum calibration, the spectral slope of each segment is determined by linear regression in valid bandwidth. Moving the bandwidth window and repeating the above procedure, the spectral slope series is converted from photoacoustic signals at each scanning position. Finally, the average slope U(x, y) at (x, y) can be calculated from the spectral slope series. The pixel value of S-mode PAT image is the average spectral slope of photoacoustic spectral.

### Data acquisition and image reconstruction software

The data were acquired with the data acquisition (DAQ) board (NI PCI-5105, National instruments). The data acquire software and computer control software were compiled using Labview (National instruments). The software for image reconstruction is developed by using a fundamental computational tool of Matlab (MathWorks).

### Materials

Uses of the human teeth in the experiments were approved by patients. The informed consent was obtained from all subjects. The experiments and methods were approved by the Institutional Review Board of Institute of Stomatological hospital, Medical School of Nanjing University. The experimental methods were carried out in accordance with the approved guidelines and regulations.

## Additional Information

**How to cite this article**: Cheng, R. *et al.* Noninvasive Assessment of Early Dental Lesion Using a Dual-Contrast Photoacoustic Tomography. *Sci. Rep.* **6**, 21798; doi: 10.1038/srep21798 (2016).

## Supplementary Material

Supplementary Information
