# Supplementary material for: Noninvasive Assessment of Early Dental Lesion Using a Dual-Contrast Photoacoustic Tomography
Source: Sci Rep. 2016 Feb 23;6:21798. doi: 10.1038/srep21798 (PMC4763185; doi:10.1038/srep21798)
Supplement: Supplementary Information [file srep21798-s1.doc]

# Supplementary Materials For

**Noninvasive Assessment of Early Dental Lesion Using a Dual-Contrast Photoacoustic Tomography**

**Renxiang Cheng^1, 3^, Jiaojiao Shao^2, 3^, Xiaoxiang Gao^1^, Chao Tao^1^, Jiuyu Ge^2^, Xiaojun Liu^1^**

1. *Key Laboratory of Modern Acoustics, Department of Physics, Collaborative Innovation Center of Advanced Microstructures, Nanjing University, Nanjing, 210093, China*
2. *Nanjing Stomatological Hospital, Medical School of Nanjing University, Nanjing, 210093, China*
3. *Equal contribution*

Correspondence and requests for materials should be addressed to C. T. (email: [taochao@nju.edu.cn](mailto:taochao@nju.edu.cn)) or J. G (email: [jyge@nju.edu.cn](mailto:jyge@nju.edu.cn)) or X. L. (email: [liuxiaojun@nju.edu.cn](mailto:liuxiaojun@nju.edu.cn)).

**Supplementary Figure:**

**Figure S1. A slice map of the sample T4.** A crack-like structure can be found inside the dentin without prolonging to the surface.


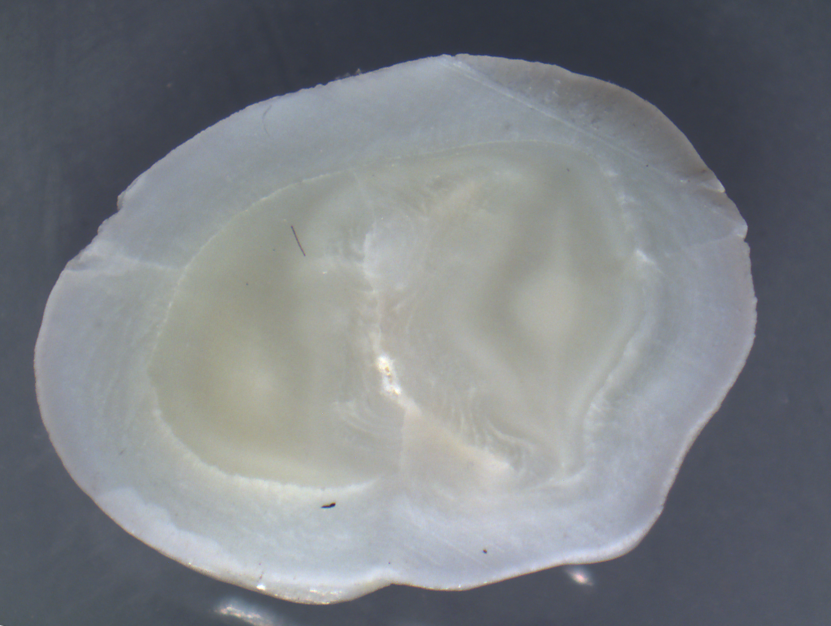


Figure S1
